# A gain-of-function NLRP3 3′-UTR polymorphism causes miR-146a-mediated suppression of NLRP3 expression and confers protection against sepsis progression

**DOI:** 10.1038/s41598-021-92547-8

**Published:** 2021-06-25

**Authors:** Furong Lu, Hongpeng Chen, Yuan Hong, Yao Lin, Lizhen Liu, Ning Wei, Qinyan Wu, Shuanglin Liao, Shuai Yang, Junbing He, Yiming Shao

**Affiliations:** 1 The Key Laboratory of Sepsis Translational Medicine, The Intensive Care Unit, The Second Affiliated Hospital of Guangdong Medical University, Minyou Road 12, Xiashan District, Zhanjiang City, 524001 Guangdong Province People’s Republic of China; 2 The Clinical Medicine Research Laboratory, The Intensive Care Unit, Jieyang Affiliated Hospital, SunYat-Sen University, Tianfu Road 107, Rongcheng District, Jieyang City, 522000 Guangdong Province People’s Republic of China; 3 The Intensive Care Unit, Affiliated Hospital of Guangdong Medical University, Zhanjiang, Guangdong People’s Republic of China; 4 The Department of Chemotherapy, Jieyang Affiliated Hospital, SunYat-Sen University, Jieyang, Guangdong People’s Republic of China; 5 Southern Marine Science and Engineering Guangdong Laboratory (Zhanjiang), Zhanjiang, 524023 Guangdong People’s Republic of China

**Keywords:** Infectious diseases, Functional genomics, Gene regulation, Genetic association study, Mutation

## Abstract

Nucleotide-binding domain and leucine-rich repeat (LRR)-containing family protein 3 (NLRP3) regulated the maturation of inflammation-related cytokines by forming NLRP3 inflammasome, which plays pivotal roles in sepsis pathogenesis. In this study, we evaluated the genetic association of NLRP3 polymorphisms with sepsis (640 patients and 769 controls) and characterized the impact of NLRP3 polymorphisms on NLRP3 expression and inflammatory responses. No significant differences were observed in genotype/allelic frequencies of NLRP3 29940G>C between sepsis cases and controls. The G allele was significantly overrepresented in patients with septic shock than those in sepsis subgroup, and the GC/GG genetypes were related to the 28-day mortality of sepsis. Lipopolysaccharide challenge to peripheral blood mononuclear cells showed a significant suppression of NLRP3 mRNA expression and release of IL-1β and TNF-α in CC compared with the GC/GG genotype category. Functional experiments with luciferase reporter vectors containing the NLRP3 3′-UTR with the 29940 G-to-C variation in HUVECs and THP-1 cells showed a potential suppressive effect of miR-146a on NLRP3 transcription in the presence of the C allele. Taken together, these results demonstrated that the 29940 G-to-C mutation within the NLRP3 3′-UTR was a gain-of-function alteration that caused the suppression of NLRP3 expression and downstream inflammatory cytokine production via binding with miR-146a, which ultimately protected patients against susceptibility to sepsis progression and poor clinical outcome.

## Introduction

Sepsis is a public health problem that is primarily caused by bacterial and fungal infections that is mediated by serious systematic inflammatory responses and leads to septic shock and multiple organ dysfunction syndrome (MODS)^1^. Even though extensive progress has been made in the development of therapeutic strategies for sepsis, it remains the main cause of mortality in intensive care units because of the extreme heterogeneity in disease outcomes possibly due to host genetic characteristics^2^. It is clear that polymorphisms in genes encoding inflammatory mediators or innate immune effectors such as IL-27, MCP-1 and ADAM17 contribute considerably to the disease course and sepsis outcome^3–5^. Thus, identifying the responsible variants will help us to elucidate genetic mechanisms that determine sepsis heterogeneity and potentially reveal new therapeutic targets for the treatment of sepsis.

In the initial stage of sepsis pathogenesis, the damage-associated molecular patterns (DAMPs) and pathogen-associated molecular patterns (PAMPs) released from microbial components and damaged tissues may interact with germline-encoded receptors called pattern recognition receptors (PRRs) and trigger chronic inflammatory responses^6^. Among these receptors, nucleotide-binding domain and leucine-rich repeat (LRR)-containing family protein 3 (NLRP3) can recruit and regulate the activation of proinflammatory caspase-1 by forming protein complexes called inflammasomes, ultimately leading to cell pyroptosis and the generation of mature forms of the inflammatory cytokines interleukin (IL)-18, IL-1β, and IL-33^7,8^. The activation of the NLRP3 inflammasome requires NLRP3 expression, which is stimulated by nuclear factor-kappa B (NF-kB) in response to exogenous and endogenous danger signals such as high glucose and bacterial lipopolysaccharide (LPS)^9,10^. The NLRP3 inflammasome has been regarded as a regulator of the pathomechanisms of various inflammation-related diseases (e.g., ulcerative colitis and sepsis), as well as a disordered host response to pathogenic infections (e.g., *S. pyogenes* and *Staphylococcus aureus*)^11–13^. Genomic deletion of NLRP3 inhibited sepsis-induced inflammatory responses and protected against sepsis in mice through the production of LXB4 and inhibition of proinflammatory cytokines^14^. In addition, NLRP3 inhibitors or gene knockout in mice resulted in increased neutrophil-dependent bacterial clearance, decreased concentrations of IL-18 and IL-1β and protection against septic lethality^15,16^. These lines of evidence demonstrated a significant role of NLRP3 in sepsis pathophysiology, and blocking NLRP3 may be a promising therapeutic strategy.

The human NLRP3 gene is located on chromosome 1q44 and contains 9 coding exons. Recently, increasing evidence has shown that genetic mutations within the key domains of the NLRP3 gene play pivotal roles in the modulation of immune inflammatory responses, ultimately resulting in a genetic predisposition to various acute and chronic inflammatory diseases^17^. Approximately 60 genetic variants within the entire NLRP3 gene have been identified so far, among which rs10754558 (29940G>C), rs35829419 (Q705K), rs10925019, rs4925648 and rs4612666 have been well studied in association with susceptibility to ulcerative colitis, Alzheimer's disease, Crohn's disease and acne vulgaris^18–21^. The 29940 G>C polymorphism within the 3′-UTR of the NLRP3 gene was significantly associated with NLRP3 transcription levels in peripheral blood mononuclear cells (PBMCs) and conferred susceptibility to ischemic stroke^22^. The gain-of-function variant Q705K within exon 3 of the NLRP3 gene generates hyperactive inflammasomes, along with the maturation and overproduction of IL-1β, leading to inflammatory damage in response to infection^23,24^. However, little is known about the clinical relationship of the NLRP3 genetic variations with sepsis susceptibility and development, and the underlying mechanisms involved in the regulatory effects of these genetic variants on sepsis-induced inflammatory processes remain to be explored.

In the present study, 640 septic patients and 769 controls were enrolled to explore the clinical relationship between the NLRP3 genetic variant 29940G>C and the susceptibility and progression of sepsis in a Han Chinese population. Mechanistic investigations in vitro were conducted to determine the impact of this functional NLRP3 polymorphism on NLRP3 transcription and sepsis-associated inflammatory responses. Our results provide a new understanding of the genetic effect of the NLRP3 inflammasome on sepsis pathophysiology and new approaches for risk assessment, as well as therapeutic strategies for sepsis patients. Besides, this study was restricted to the Chinese Han population with small sample size. Thus our results may not be generalized to other populations, and need to be further validated in a larger and multiethnic population of subjects.

## Material and methods

### Study population

This multi-center case–control study enrolled 640 sepsis patients in the intensive care unit (ICU) and 769 healthy individuals in the Health Examination Center between May 2017 and November 2019 at the same period time, among whom 286 patients and 321 controls were from Affiliated Hospital of Guangdong Medical University (Zhanjiang, southern China), 185 patients and 188 controls were from the Fourth Affiliated Hospital of Harbin Medical University (Haerbin, Northern China), 101 patients and 124 controls were from the Central Hospital of Wuhan (Wuhan, Central China), 68 patients and 136 controls were from the Jieyang People's Hospital and Longgang District People’s Hospital of Shenzhen (Jieyang and Shenzhen, southern China). The diagnosis of sepsis and septic shock was defined in accordance with the Third International Consensus Definitions for Sepsis and Septic Shock (Sepsis-3)^25^. The septic patients and healthy controls who met the exclusion criteria were excluded according to our previous studies^3,26^. The clinical variables such as gender, age, blood microbiological cultures, source of infection, Sequential Organ Failure Assessment (SOFA) and APACHE II score were collected. Once the diagnoses were established, the peripheral blood from septic patients was collected within 12 h. The ethics committee of Affiliated Hospital of Guangdong Medical University approved this study. The studied participants or their families wrote the informed consent, and all the research procedures met the standards of the 1964 Helsinki declaration.

### Genotyping of NLRP3 polymorphism

TIANamp Blood DNA kit (Tiangen Biotech, Beijing, China) was applied to prepare genomic DNA from blood leukocytes of sepsis patients and healthy individuals. The iMLDR™ multiple SNP typing kit (Shanghai Tanhao biotechnology co., LTD) was applied to genotype the NLRP3 genetic variant rs10754558. The experimental results collected from the sequencer were analysed by GeneMapper 4.0 (Applied Biosystems, USA). Ten percent of samples were randomly selected for re-examination to verify the accuracy.

### Preparation of PBMCs for cell culture and LPS stimulation

A total of 50 healthy controls were randomly selected for isolation of PBMCs. After isolation from the peripheral blood by Lymphoprep™ (Axis-Shield PoCAS, Oslo, Norway), the PBMCs were stored at − 80 °C until used for the in vitro LPS-stimulated experiments. RPMI-1640 medium (Thermo Fisher Scientific) was used for cell culture. Approximately 5 × 10^5^ cells with 500 ng/mL LPS were cultured in 12-well plates (n = 25), and treatment with PBS vehicle was set as the control (n = 25). After 8 h of incubation, cells were separated for qRT-PCR analysis and supernatants were separated for cytokine measurements.

### Quantitative real-time polymerase chain reaction (qRT-PCR)

For qRT-PCR analysis of NLRP3 expression, PBMCs from 100 randomly selected subjects (50 patients and 50 controls) or LPS stimulation experiments in vitro were used for RNA extraction by Trizol reagent (Sangon Biotech). RevertAid™ First Strand cDNA Synthesis Kit (Thermo Fisher Scientific) was used for preparation of cDNA from RNA. SYBR Green mix (Takara) was used for reaction in a TL988 real-time PCR system (TianLong, Xi’an, China). The gene-specific primers synthesized by Sangon Biotech (China) were used as follows: NLRP3, 5′-TGTCGGGAGGTGAGCCTTGTG-3′ and 5′-GATCTTGTGGATGGGTGGGTTTGG-3′; miR-146a, 5′-CTGCCGCTGAGAACTGAATT-3′ and 5′-CAGAGCAGGGTCCGAGGTA-3′; GAPDH, 5′-TGGTGAAGACGCCAGTGGA-3′ and 5′-GCACCGTCAAGGCTGAGAAC-3′; U6, 5′-AACGCTTCACGAAT TTGCGT-3′ and 5′-CTCGCTTCGGCAGCACA-3′. The expression of NLRP3 and miR-146a calculate with the 2^−△△CT^ method was normalized to GAPDH and U6, respectively.

### Western blot analysis

The PBMCs isolated from 18 sepsis patients and 6 healthy controls were lysed in RIPA buffer with protease/phosphatase inhibitors (Beyotime, Shanghai, China), and the concentration of protein was detected using a BCA Protein Assay Kit (Beyotime, Shanghai, China). The protein was separated and then transferred onto polyvinylidene difluoride membranes (Millipore, Bedford, MA, USA). The membranes were incubated with antibodies against NLRP3/Cryopyrin (sc-134306; Santa Cruz, CA, USA) and β-actin (Beyotime, Shanghai, China) at 4 °C overnight, followed by HRP-linked secondary antibody. Finally, the protein bands were visualized using a BeyoECL Star kit (Beyotime, Shanghai, China).

### Cell culture and transfection

Human umbilical vein endothelial cells (HUVECs) and THP-1 cells were obtained from Shanghai Institute of Cell Biology. The cells were incubated in RPMI-1640 medium supplemented with FBS (10%) and penicillin/streptomycin (1%). Lentivirus (LV) packaging of LV3-miR-146a (miR-146a overexpression) with sequence 5′-TGAGAACTGAATTCCATGGGTT-3′, LV3-miR-580-3p (miR-580-3p overexpression) with sequence 5′-TTGAGAATGATGAATCATTAGG-3′, LV3-miR-589-5p (miR-589-5p overexpression) with sequence 5′-TGAGAACCACGTCTGCTCTGAG-3′ and LV3-NC (negative control) with sequence 5′-TTCTCCGAACGTGTCACGT-3′ was carried out by GenePharma (Shanghai, China). The cells were infected by LV3-miR-146a, LV3-miR-580-3p or LV3-miR-589-5p to establish stable expression cell strain, and infection with LV3-NC was set as the control cells. After 48 h of infection, qRT-PCR analysis was carried out to determine the miR-146a, miR-580-3p and miR-589-5p expression.

### RNA secondary structure analysis and 3′-UTR luciferase assays

A 439-bp mRNA in the 3′UTR of NLRP3 containing rs10754558 G-to-C mutant was submitted to the RNAfold Web Server (http://rna.tbi.univie.ac.at/cgi-bin/RNAfold.cgi) setting default parameters to produce positional entropies, centroid structures and Potential minimum free energy (MFE) structures. Next, a fragment with 313 bp length in the 3′UTR region of NLRP3 containing rs10754558 G-to-C mutant was inserted downstream to luciferase reporter gene in pmirGLO luciferase reporter vectors. Lipofectamine 3000 (Invitrogen, MD, USA) was used for cell transfection, and dual-luciferase assay kit (Beyotime, Shanghai, China) was used to measure the activities of luciferase in a SpectraMax L Chemiluminescent reader (Molecular Devices, USA).

### Enzyme linked immunosorbent assay (ELISA)

The IL-1β, IL-6, and TNF-α concentrations in supernatants separated from studied subjects plasma or cell culture medium were detected by each specific ELISA kit (Boster Biological Technology Co., Ltd., Wuhan, China). Infinite F50 microplate reader (Tecan, Switzerland) was used to detect the absorbance at 450 nm.

### Data analysis and statistics

GraphPad Prism 6.0 software (GraphPad Software Inc., San Diego, CA, USA) was applied to data analysis. Fisher’s exact test or Chi-squared test was performed to calculate the genotypes and allelic distributions of NLRP3 29940G>C polymorphism, and the false discovery rate was evaluated by Benjamini–Hochberg procedure for multiple testing correction. QUANTO 1.2 software was applied to power analysis, which exhibited 99.7% power at a significance level of 0.05 and an odds ratio of 1.5 for the 29940 G>C polymorphism based on the sample size. The 28-day ICU survival curve was drawn by using the log-rank test and Kaplan–Meier method. The data was presented as the mean ± SEM and compared using nonparametric Mann–Whitney U test or Student’s t-test. A p value < 0.05 was set as statistical significance.

## Results

### The characteristics of the study population

The demographic characteristics of the studied subjects are presented in Table 1. No significant differences were observed concerning sex (*p* = 0.204) or age (*p* = 0.092) between sepsis patients (n = 640) and healthy controls (n = 769). The common pathogens identified in this study population were *Acinetobacter baumannii* (25.5%) and *Pseudomonas aeruginosa* (12.5%). The primary sources of infection were the respiratory tract infection (64.5%), abdominal infection (27.0%) and primary bloodstream infection (13.8%). The patients who suffered sepsis and septic shock accounted for 354 (55.3%) and 286 (44.7%), respectively. The 28-day ICU mortality rate was 25.8% in this study.

**Table 1:** Clinical characteristics of sepsis patients and healthy controls.

Variable	Sepsis (n = 640) Number (%)	Control (n = 769) Number (%)	p
**Demographics**			
Age (years), mean ± SEM	61.9 ± 0.7	60.4 ± 0.6	0.092
Gender, male/female, n	422/218	482/287	0.204
**Sepsis status, n (%)**			
Sepsis subtype	354 (55.3)	N.A	
Septic shock	286 (44.7)	N.A	
**Source of infection, n (%)**			
Respiratory tract infection	413 (64.5)	N.A	
Primary bloodstream infection	88 (13.8)	N.A	
Abdominal infection	173 (27.0)	N.A	
Urinary tract infection	69 (10.8)	N.A	
Catheter-associated infection	48 (7.5)	N.A	
Brain	47 (7.3)	N.A	
trauma	47 (7.3)	N.A	
Others	48 (7.5)	N.A	
**Infection types, n (%)**			
Gram-positive	97 (15.2)	N.A	
Gram-negative	335 (52.3)	N.A	
Mixed Gram-negative and -positive	201 (31.4)	N.A	
Fungus	143 (22.3)	N.A	
Negative blood culture	62 (9.7)	N.A	
**Pathogenic bacteria, n (%)**			
Acinetobacter baumannii	163 (25.5)	N.A	
Monilia albican	59 (9.2)	N.A	
Yeast sample sporphyte	39 (6.1)	N.A	
Aspergillus	25 (3.9)	N.A	
*Klebsiella pneumoniae*	45 (7.0)	N.A	
*Pseudomonas aeruginosa*	80 (12.5)	N.A	
*Staphylococcus aureus*	53 (8.3)	N.A	
*Escherichia coli*	74 (11.6)	N.A	
Others	111 (17.3)	N.A	
SOFA score (mean ± SEM)	9.1 ± 0.2	N.A	
APACHE II score (mean ± SEM)	26.8 ± 4.5	N.A	
28-day mortality, n (%)	165 (25.8)	N.A	

*N.A* not applicable, *SOFA* Sequential Organ Failure Assessment, *APACHE II* Acute Physiology and Chronic Health Evaluation II.

### The association of NLRP3 29940G>C polymorphism with sepsis susceptibility and progression

The genotypes and allelic distributions of the NLRP3 29940G>C polymorphism were consistent with the Hardy–Weinberg equilibrium in sepsis patients and healthy controls (All *p* > 0.05; Supplementary Table S1). As shown in Table 2, no significant differences in the genotype/allelic frequencies of the 29940G>C polymorphism were found between sepsis cases and healthy individuals (all *p* > 0.05). When the cases were separated into sepsis and septic shock, the data showed that patients with the GG/GC genotypes were associated with a significantly increased risk of progression from sepsis to septic shock (GG/GC vs. CC: *p* = 0.005, OR = 1.608, 95% CI 1.152–2.245, Table 3). Similarly, G allele carriers had an increased risk of sepsis progression (G allele vs. C allele: *p* = 0.003, OR = 1.402, 95% CI 1.120–1.754). These significant differences were still observed after the Benjamini–Hochberg correction.

**Table 2:** Genotype and allele frequencies distribution of the NLRP3 rs10754558 in the patients with sepsis and healthy controls.

rs10754558	Controls n (%)	Patients n (%)	p	p*	Odds ratio (95% CI)
GG	127 (16.5)	105 (16.4)	0.513	0.684	–
GC	398 (51.8)	314 (49.1)	–	–	–
CC	244 (31.7)	221 (34.5)	–	–	–
GG/GC	525 (68.3)	419 (65.5)	0.266	0.684	1.135 (0.908, 1.418)
GC/CC	642 (83.5)	535 (83.6)	0.956	0.956	0.992 (0.748, 1.317)
G	652 (42.4)	524 (40.9)	0.435	0.684	0.942 (0.810, 1.095)
C	886 (57.6)	756 (59.1)	–	–	1.000 (reference)

*95% CI* 95% confidence interval.

*False discovery rate-adjusted p-value for multiple hypotheses testing using the Benjamini–Hochberg method.

**Table 3:** Genotype and allele frequencies distribution of the NLRP3 rs10754558 in the different sepsis status.

rs10754558	Sepsis subtype n (%)	Septic shock n (%)	p	p*	Odds ratio (95% CI)
GG	49 (13.8)	56 (19.6)	0.011	0.015	–
GC	166 (46.9)	148 (51.7)	–	–	–
CC	139 (39.3)	82 (28.7)	–	–	–
GG/GC	215 (60.7)	204 (71.3)	0.005	0.010	1.608 (1.152, 2.245)
GC/CC	305 (86.2)	230 (80.4)	0.051	0.051	0.660 (0.434, 1.004)
G	264 (37.3)	260 (45.5)	0.003	0.012	1.402 (1.120, 1.754)
C	444 (62.7)	312 (54.5)	–	–	1.000 (reference)

*95% CI* 95% confidence interval.

*False discovery rate-adjusted p-value for multiple hypotheses testing using the Benjamini–Hochberg method.

### The association of the NLRP3 29940G>C polymorphism with the mortality of septic patients

**Figure 1:**
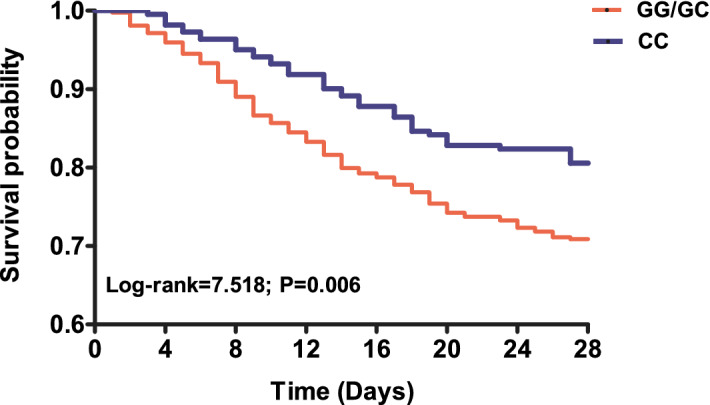
Kaplan–Meier survival analysis of septic patients. The influence of NLRP3 29940G>C polymorphism on the 28-day survival of patients with sepsis was evaluated via using the Kaplan–Meier survival analysis.

We further evaluated the effect of NLRP3 29940G>C polymorphism on the 28-day mortality of septic patients. As presented in Table 4, the frequencies of the G allele and GG/GC genotypes in 28-day non-surviving sepsis patients were significantly overrepresented compared to those in the surviving sepsis patients (GG/GC vs. CC: *p* = 0.008, OR = 1.700, 95% CI 1.146–2.522; G allele vs. C allele: *p* = 0.010, OR = 1.395, 95% CI 1.083–1.795). These differences still exhibited statistically significance after the Benjamini–Hochberg multiple-testing corrections (GG/GC vs. CC: *p* = 0.032; G allele vs. C allele: *p* = 0.020). Furthermore, Kaplan–Meier survival analysis showed that 28-day survival exhibited much worse in septic patients with the GG/GC genotypes than in septic patients carrying the CC genotype (log-rank = 7.518, *p* = 0.006; Fig. 1).

**Table 4:** Genotype and allele frequencies distribution of the NLRP3 rs10754558 in the 28-day surviving and non-surviving sepsis patients.

rs10754558	Survivors n (%)	Non-survivors n (%)	p	p*	Odds ratio (95% CI)
GG	72 (15.1)	33 (20.0)	0.024	0.032	–
GC	225 (47.4)	89 (53.9)	–	–	–
CC	178 (37.5)	43 (26.1)	–	–	–
GG/GC	297 (62.5)	122 (73.9)	0.008	0.032	1.700 (1.146, 2.522)
GC/CC	403 (84.8)	132 (80.0)	0.148	0.148	0.715(0.453, 1.128)
G	369 (38.8)	155 (47.0)	0.010	0.020	1.395 (1.083, 1.795)
C	581 (61.2)	175 (53.0)	–	–	1.000 (reference)

*95% CI* 95% confidence interval.

*False discovery rate-adjusted p-value for multiple hypotheses testing using the Benjamini–Hochberg method.

### Effect of the NLRP3 29940G>C polymorphism on NLRP3 expression and cytokine production

**Figure 2:**
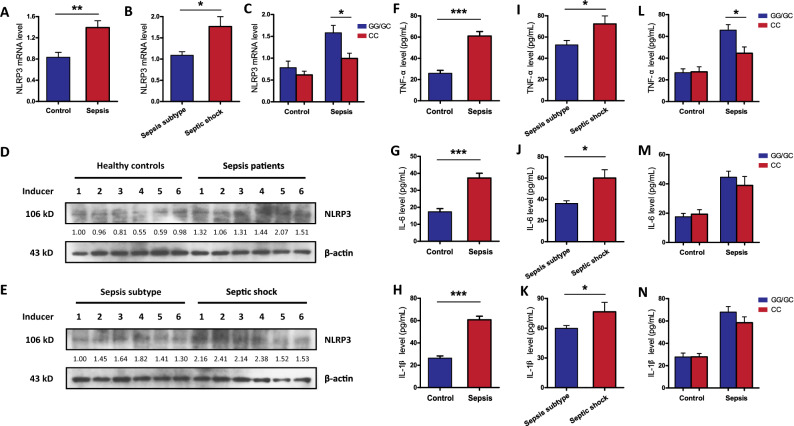
The expression of NLRP3 gene and its downstream inflammatory cytokines in the studied subjects. (**A**–**C**) The mRNA expression level of NLRP3 in septic patients (n = 50) and healthy controls (n = 50) with different rs10754558 polymorphism; (**D**, **E**) the production of NLRP3 protein in healthy controls and septic patients were detected by western blot. Full-length blots are presented in the Supplementary Fig. S2; The serum levels of inflammatory cytokines TNF-α (**F**), IL-6 (**G**) and IL-1β (**H**) in sepsis cases and controls; Comparison of serum TNF-α (**I**), IL-6 (**J**) and IL-1β (**K**) levels in different sepsis status (sepsis subject and septic shock); Comparison of serum TNF-α (**L**), IL-6 (**M**) and IL-1β (**N**) levels in subjects with different genotypes at the NLRP3 rs10754558 site. Values of relative expression levels are shown as means ± SEM. *p < 0.05, **p < 0.01, ***p < 0.001.

A total of 50 septic patients and 50 healthy controls were randomly selected to evaluate the potential association between the NLRP3 29940G>C polymorphism and the expression of NLRP3 and related inflammatory cytokines. As presented in Fig. 2, sepsis patients exhibited significantly higher expression and production of NLRP3 and the proinflammatory cytokines (TNF-α, IL-6 and IL-1β) than healthy controls, and these levels were significantly increased with sepsis progression. When sepsis cases were separated into subgroups by genotype, carriers of the GG/GC genotypes at the NLRP3 29940G>C site exhibited significantly higher NLRP3 mRNA and TNF-α levels than carriers with the CC genotype. No significant differences in NLRP3 and cytokine levels were found in healthy controls with different genotypes.

**Figure 3:**
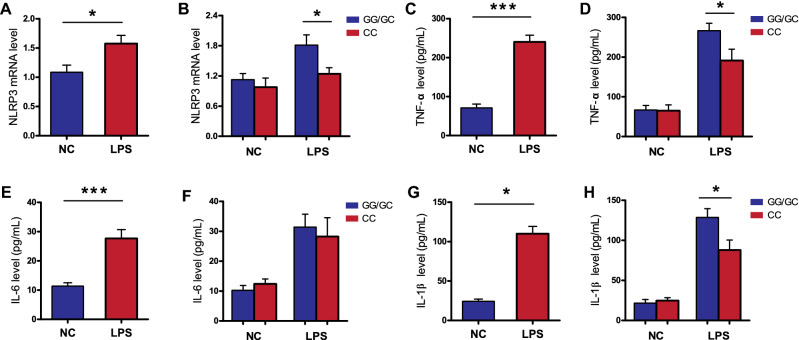
The rs10754558 polymorphism is associated with increased expression of NLRP3 gene and its downstream inflammatory cytokines in LPS-stimulated peripheral blood mononuclear cells (PBMCs). The PBMCs isolated from 50 healthy subjects with different rs10754558 genotypes were treated with 500 ng/mL of LPS (n = 25) and PBS (n = 25) as negative control. (**A**, **B**) The expression of NLRP3 were detected using qRT-PCR; The supernatant concentrations of TNF-α (**C**, **D**), IL-6 (**E**, **F**) and IL-1β (**G**, **H**) were measured by ELISA. *LPS* lipopolysaccharide, *qRT-PCR* quantitative real-time polymerase chain reaction, *ELISA* enzyme-linked immunosorbent assay. Values of relative expression levels are shown as mean ± SEM. *p < 0.05, ***p < 0.001.

To further validate whether the NLRP3 29940G>C polymorphism was likely influence NLRP3 expression at the transcriptional level, qRT-PCR analysis was conducted in LPS-stimulated PBMCs from 50 healthy controls to demonstrate the connection between genotype and phenotype. Our results showed that the NLRP3 29940G>C variant was closely associated with the responsiveness of PBMCs to LPS stimulation. LPS stimulation induced significantly higher NLRP3 expression and proinflammatory cytokine (IL-1β and TNF-α) production in PBMCs with the 29940GG/GC genotypes compared with those with the CC genotype, while these differences were not observed without LPS stimulation (Fig. 3). However, no significant difference in IL-6 production was observed between PBMCs with different 29940G>C genotypes.

### Influence of the NLRP3 3′-UTR polymorphism 29940G>C on NLRP3 mRNA stability

**Figure 4:**
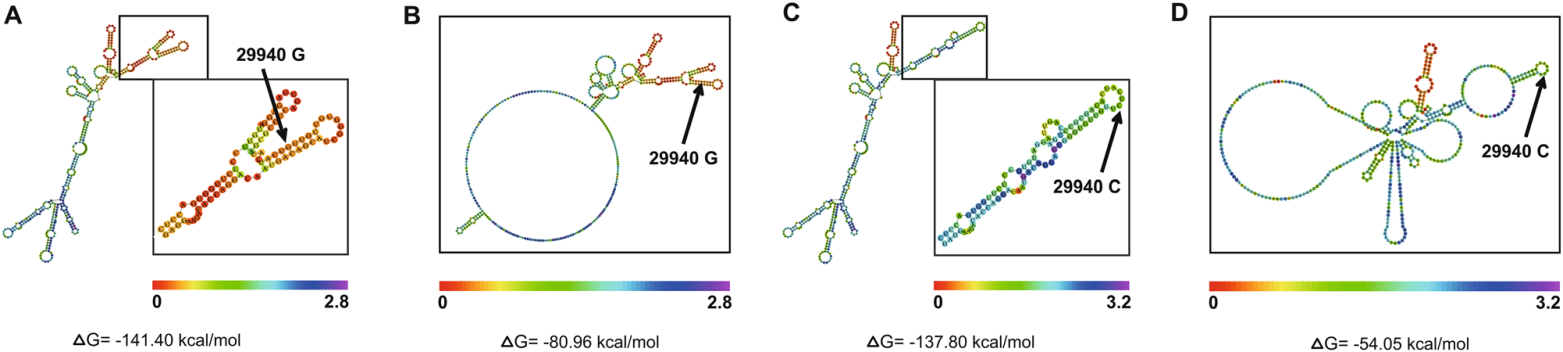
Prediction analysis of NLRP3 mRNA secondary structure with RNAfold Web Server. (**A**, **B**) showed MFE positional and centroid positional entropy, respectively, for the 29940 G allele; (**C**, **D**) showed MFE positional and centroid positional entropy. The higher MFE and centroid MFE values of the NLRP3 mRNA secondary structure with 29940 C mutant indicated less stable mRNA secondary structure.

Considering the location of the 29940G>C polymorphism within the 3′-UTR of the NLRP3 gene, we further examined the effect of the sepsis-associated G-to-C mutation at the rs10754558 site on NLRP3 mRNA stability. As shown in Fig. 4, NLRP3 mRNA secondary structure prediction analysis with the RNAfold Web Server indicated that the MFE value in the 3′-UTR of NLRP3 with the G-to-C mutation at the rs10754558 site had a notable variation from ∆G = − 141.40 to − 137.80 kcal/mol. The centroid secondary structure MFE value in this predicted mRNA secondary structure with the G allele of the 29940G>C polymorphism (∆G = − 80.96 kcal/mol) was also observed to be lower than that with the C allele (∆G = − 54.05 kcal/mol). This G-to-C mutation in the internal base pairing contributed to markedly different bubble and stem-loop structures, ultimately leading to a possible decrease in NLRP3 mRNA stability.

**Figure 5:**
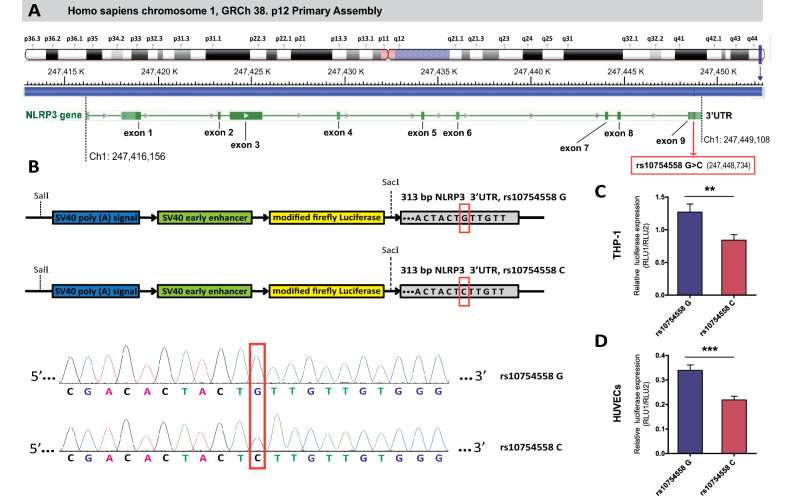
The location of functional rs10754558 polymorphism in NLRP3 gene and its effect on the post-transcriptional regulation of NLRP3 expression. (**A**) The human NLRP3 gene is encoded in Homo sapiens chromosome 1 q44 (247416156–247449108) containing 9 coding exons (shown as bottle green bars), and the rs10754558 polymorphism within 3′UTR of NLRP3 gene is located at chromosome 1 (247448734); (**B**) A fragment with 313 bp length in the 3′UTR region of NLRP3 containing rs10754558 G-to-C mutantion was amplified and cloned into pmirGLO luciferase reporter vectors; (**C**, **D**) After 48-h of transfection, the transcriptional enhancer activities of rs10754558 in HUVEC and THP-1 cells were detected using dual-luciferase report assays. Firefly luciferase signals were normalized with Renilla luciferase signals. Bar graphs indicate the mean ± SEM for a minimum of three independent experiments. **p < 0.01, ***p < 0.001.

The human NLRP3 gene containing nine coding exons is located on chromosome 1q44 (247416156–247449108), and the 29940G>C polymorphism within 3′-UTR of NLRP3 gene was positioned at chromosome 1 (247448734) according to the GRCh 38. p12 Primary Assembly (Fig. 5A). Next, a luciferase assay with pmirGLO luciferase reporter vectors containing the G-to-C mutation at the rs10754558 site (Fig. 5B) was conducted in HUVECs and THP-1 cells to gain further insights into the functional impact of this NLRP3 3′-UTR polymorphism on NLRP3 expression. The pmirGLO luciferase reporter vector with the NLRP3 29940G allele showed significantly higher activities than the vector with the C allele (Fig. 5C,D).

### NLRP3-29940G>C is a gain-of-function alteration that suppresses NLRP3 expression by altering miR-146a-5p binding

**Figure 6:**
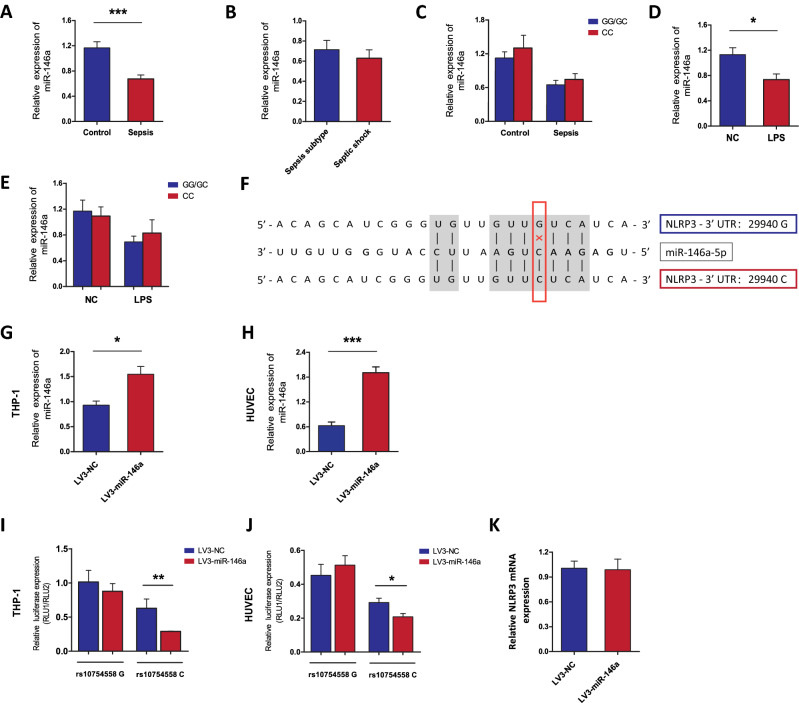
The NLRP3-29940G>C is a gain-of-function alteration causing suppression of NLRP3 expression via altering miR-146a-5p binding. (**A**–**C**) The miR-146a expression in septic patients (n = 50) and healthy controls (n = 50) with different rs10754558 polymorphism; (**D**, **E**) PBMCs isolated from 50 healthy subjects with different rs10754558 genotypes were stimulated with 500 ng/mL of LPS (n = 25) or PBS (n = 25) in vitro, and then miR-146a expression was detected; (**F**) Bioinformatics predicted that the rs10754558 G-to-C mutation was located at the binding site between 3′UTR of NLRP3 and miR-146a-5p; (**G**, **H**) HUVECs and THP-1 cells were infected by LV3-miR-146a (miR-146a overexpression) or LV3-NC, and the infection efficiency of LV3-miR-146a was determined by qRT-PCR analysis; (**I**, **J**) The effect of miR-146a on the transcriptional activities of rs10754558 G>C in HUVECs and THP-1 cells were determined by dual-luciferase report assays; (**K**) The effect of miR-146a on NLRP3 expression in THP-1 cells was determined by qRT-PCR analysis. Bar graphs indicate the mean ± SEM for a minimum of three independent experiments. *p < 0.05, **p < 0.01, ***p < 0.001.

As the 29940G>C polymorphism was located in the 3′-UTR of the NLRP3 gene, we focused on its effect on gene interactions with miRNAs. Bioinformatics analyses predicted by miRNASNP-v3 (http://bioinfo.life.hust.edu.cn/miRNASNP/) and TargetScan (http://www.targetscan.org/) revealed a target binding site of miR-146a-5p enabled by the 29940 G-to-C mutation within the 3′-UTR of the NLRP3 gene (Fig. 6)^27^. We next detected the miR-146a expression in 50 septic patients and 50 healthy controls. Our results showed that sepsis patients exhibited significantly lower expression level of miR-146a compared to the healthy controls (Fig. 6A). No significant differences in miR-146a expression were observed in patients with different types of sepsis or different genotypes of rs10754558 polymorphism (Fig. 6B,C). Similarly, in vitro LPS stimulation induced decreased expression of miR-146a in the PBMCs isolated from 50 healthy subjects, while differential levels of miR-146a were not detected in PBMCs with different genotypes of NLRP3 polymorphism (Fig. 6D,E).

To validate the potential role of miR-146a-5p in the attenuation of NLRP3 expression mediated by the NLRP3 29940 G-to-C mutation, HUVECs and THP-1 cells were infected with LV3-miR-146a (miR-146a overexpression vector) and LV3-NC (negative control) to establish a stably overexpressing cell line (Fig. 6F–H). Next, pmirGLO-NLRP3-3′-UTR-29940G and pmirGLO-NLRP3-3′-UTR-29940C were transfected into the cells for 48 h. As shown in Fig. 6I,J, miR-146a-5p significantly decreased the luciferase reporter activity of the pmirGLO-NLRP3-3′-UTR-29940C vector, while no obvious variations were found in the pmirGLO-NLRP3-3′-UTR-29940G vector. With regards to the other miRNAs predicted by miRNASNP-v3 and TargetScan, neither miR-580-3p nor miR-589-5p overexpression exerted a significant effect on the activities of the luciferase reporter vectors containing the NLRP3 3′-UTR with 29940 G-to-C variation in THP-1 cells (Supplementary Fig. S1). These results indicated that NLRP3-29940G>C is a gain-of-function alteration that causes the suppression of NLRP3 expression by altering the binding of miR-146a-5p to the 3′-UTR of NLRP3. However, no significant difference in NLRP3 expression was observed between THP-1 cells infected with LV3-NC and LV3-miR-146a (Fig. 6K).

## Discussion

It has been demonstrated that inappropriate systemic inflammatory responses contribute to the poor prognosis of sepsis patients^28^. A number of publications have indicated that a significant portion of the variability in predisposition to the occurrence and progression of sepsis was potentially attributed to genetic factors, particularly certain functional genetic variants^29^. Increasing evidence from our previous studies and others has revealed an important effect of genetic variations in at least 40 genes encoding inflammation-related factors on interindividual differences in the immune inflammatory response and susceptibility to sepsis outcomes^30–32^. In this study, we explored the clinical relevance of NLRP3 genetic variation, rs10754558 (29940G>C), to sepsis susceptibility and progression in a Han Chinese population (640 septic patients and 769 controls) for the first time. Our data indicated that patients with the 29940 GG/GC genotypes exhibited significantly increased risk of progression from sepsis to septic shock, and the 28-day survival in patients with sepsis carrying the GG/GC genotypes exhibited much worse than in the CC genotype carriers, which indicated that the 29940G>C may act as a risk factor for the development of sepsis and poor clinical outcome. Furthermore, functional experiments suggested that this sepsis-associated NLRP3-29940G>C polymorphism might be a gain-of-function alteration that suppresses NLRP3 expression and downstream cytokine production by altering the binding of miR-146a-5p to the 3′-UTR of NLRP3.

NOD-like receptors (NLRs) are pattern recognition receptors that recognize various pathogens and damage-associated molecular patterns and are important in the context of sepsis^33^. Among these receptors, NLRP3 (also known as NALP3 and cryopyrin) forms a cytosolic signaling complex termed the NLRP3 inflammasome, which is an important molecular platform that controls the activation of proinflammatory caspase-1 and the maturation of the potent inflammatory mediators IL-18 and IL-1β, which play pivotal roles in sepsis pathogenesis^9^. The expression of NLRP3 inflammasome mRNA is significantly upregulated in patients with sepsis and various murine models of sepsis and is closely associated with the disease course and the clinical prognosis of sepsis patients^34,35^. Consistently, our data showed that sepsis patients exhibited significantly higher NLRP3 gene expression and protein production than healthy volunteers, and these levels were also increased with disease severity. These results verified the pivotal involvement of NLRP3 in the clinical course of sepsis as a proinflammatory regulator, as well as an indicator of disease severity.

Several studies have paid close attention to in-depth genetic analysis of the NLRP3 gene in various inflammation-related diseases^18–21^. Herrmann et al*.* discovered that the rs7525979 C-to-T mutation in the NLRP3-coding region conferred protection against susceptibility to Parkinson’s disease by impacting NLRP3 translation efficiency^36^. The 29940 G>C genetic variation in the 3′-UTR of NLRP3 attenuated the stability and expression of NLRP3 mRNA in a gain-of-function manner, which resulted in susceptibility to several inflammation-related disorders, such as asthma, ischemic stroke and rheumatoid arthritis^22,37,38^. Other studies have demonstrated that the Q705K genetic variation in exon 3 of the NLRP3 gene is also a gain-of-function mutation that generates overactive NLRP3 inflammasomes and overproduction of IL-18, IL-1β and TNF-α, which are closely associated with atopic dermatitis, ulcerative colitis and abdominal aortic aneurysms^39,40^. However, little is known about the clinical relevance of the gain-of-function variant 29940 G>C to the susceptibility and progression of sepsis.

The initial results in this study showed no statistically significant differences in the genotype/allelic frequencies of the NLRP3 29940G>C variation between sepsis patients and healthy volunteers, suggesting that there was no effect on the onset of sepsis. Novel findings from additional stratification analyses showed that the frequencies of the G allele and the GG/GC genotypes in the septic shock subgroup were significantly overrepresented compared to those in the sepsis subgroup. Carriers of 29940 GG/GC exhibited significantly increased NLRP3 mRNA expression compared to that of the CC genotype carriers, indicating a role of the 29940 G>C polymorphism in the susceptibility to sepsis progression by altering NLRP3 gene transcription. As the mRNA secondary structure is an important feature of the functional efficiency of cis-acting factors in the 3′-UTR, we examined the impact of the 29940 G-to-C mutation in the NLRP3 3′-UTR on NLRP3 mRNA stability. RNA secondary structure prediction indicated that the G-to-C mutation in the internal base pairing contributed to markedly different bubble and stem-loop structures and an increased MFE, ultimately leading to a possible decrease in NLRP3 mRNA stability. Next, a luciferase reporter assay was conducted to evaluate the functional impact of the 29940 G-to-C mutation on NLRP3 expression and showed that the allele-specific luciferase vector with the sepsis risk-associated G allele exhibited significantly higher activities than the C allele, which corroborated the findings of several previous studies^22,37^. To further confirm these results, we performed in vitro LPS stimulation experiments to verify the impact of the 29940 G-to-C mutation on NLRP3 mRNA expression. As predicted, LPS-induced NLRP3 gene expression was significantly increased in PBMCs with the sepsis-associated risk GG/GC genotypes compared with the CC genotype. However, no significant differences in NLRP3 expression or cytokine production were observed in PBMCs with different genotypes in the absence of LPS stimulation. These results suggested that the hereditary effects of the NLRP3 29940 G-to-C mutation made a real difference in the response to exogenous and endogenous danger signals such as bacterial LPS, which predisposed certain sepsis patients to disease progression from sepsis to sepsis shock rather than the occurrence of sepsis.

MiRNAs are small noncoding RNAs that bind to specific mRNA molecules and cause mRNA degradation or suppression of target gene expression, and they play pivotal roles in the modulation of multiple cellular processes, such as proliferation, differentiation, inflammation and apoptosis^41^. Increasing evidence has demonstrated the important impact of specific genetic variations within the 3′-UTR on the binding of miRNAs to cause the gain or loss of regulation by certain miRNAs, ultimately resulting in susceptibility to multiple diseases^42,43^. According to bioinformatics analyses predicted by miRNASNP-v3 (http://bioinfo.life.hust.edu.cn/miRNASNP/) and a previous study^27,38^, the 29940 G-to-C mutation in the 3′-UTR of NLRP3 might establish a new functional binding site for miR-146a-5p, which contributes to potential miR-146-5p recruitment and binding. Research so far has demonstrated an anti-inflammatory effect of miR-146a-5p on the regulation of multiple signaling pathways involved in the modulation of various inflammation-related cytokines^44^. Our results showed that miR-146a-5p expression was significantly downregulated under a sepsis or in vitro LPS stimulation, in accordance with the findings of our previous study^45^. However, no significant differences in miR-146a expression were observed in patients with different types of sepsis or different genotypes of NLRP3 polymorphism. The luciferase reporter experiment showed a significantly suppressive effect of miR-146a on NLRP3 transcription in the presence of the mutant-type C allele in HUVEC and THP-1 cells, while these differences were not observed in the presence of the wild-type G allele. These results further validated that the 29940 G>C polymorphism in the 3′-UTR of the NLRP3 gene was a gain-of-function alteration causing the suppression of NLRP3 expression by miR-146a-5p, which conferred protection against progression from sepsis to septic shock. However, we failed to detect the inhibitory effect of miR-146a on NLRP3 expression in a monocytic THP-1 cell line, which might be attributed to the wild-type G allele at rs10754558 site in THP-1 cells. Our further study would explore this gain-of-function mutation in NLRP3 gene by constructing the NLRP3 gene point mutation cell line via CRISPR/Cas9 technology.

**Figure 7:**
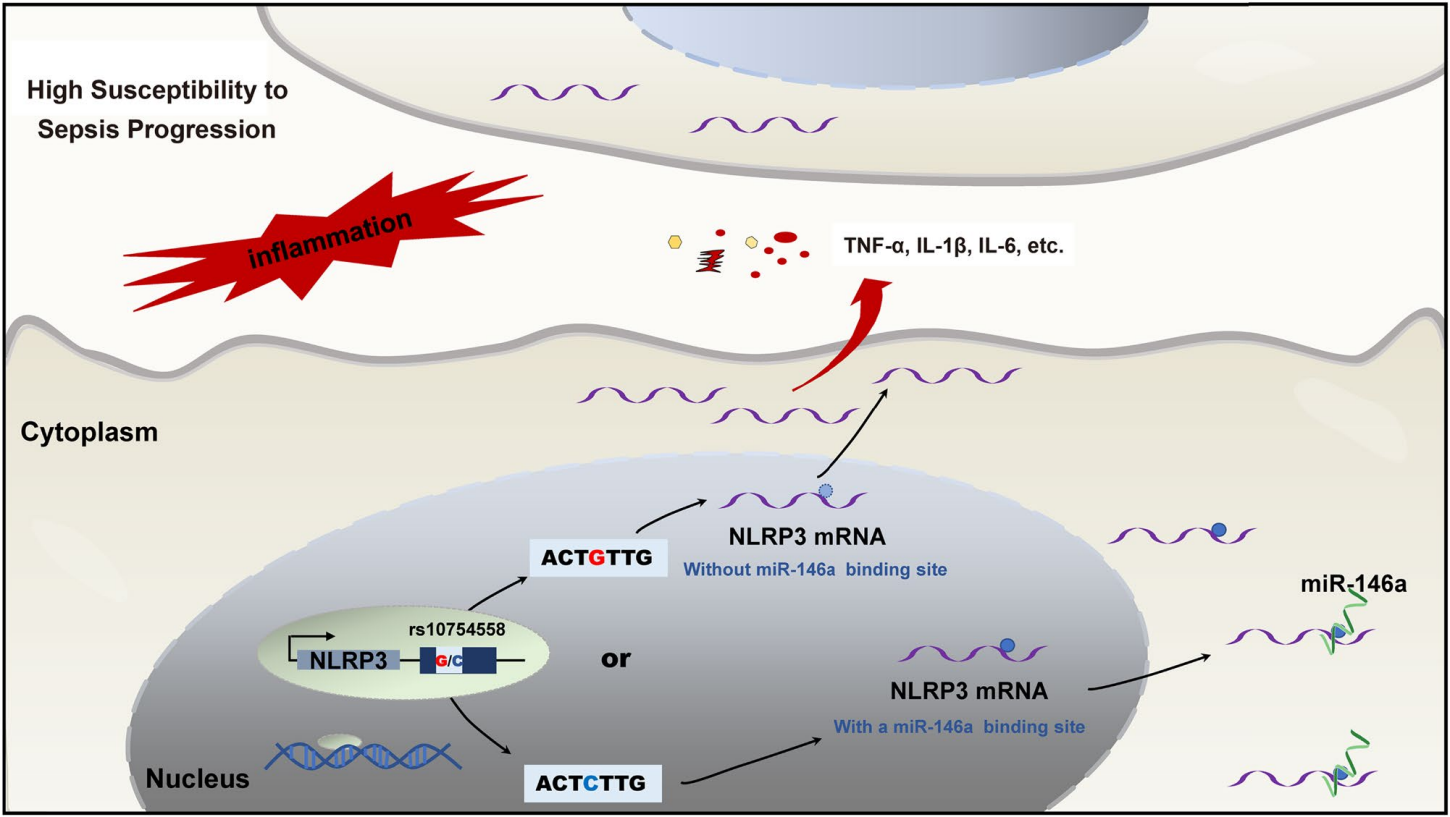
The molecular mechanism of NLRP3 3′-UTR polymorphism-mediated miR-146a-5p modulation on NLRP3 expression in the progression of sepsis. The 29940 G-to-C mutation within the NLRP3 3′-UTR was a gain-of-function alteration that caused the suppression of NLRP3 expression and downstream inflammatory cytokine production via binding with miR-146a-5p, which ultimately protected patients against susceptibility to sepsis progression.

Upon sensing PAMPs and DAMPs, NLRP3 assembles a group of high-molecular-weight protein complexes that can activate caspase 1-mediated release of bioactive proinflammatory cytokines, as well as nonclassical secretion of various cytosolic proteins, causing excessive inflammatory responses and pyroptosis^7^. NLRP3 activation contributes to the cascade release of inflammatory cytokines, including IL-33, IL-18, IL-1β and HMGB1, and prostaglandins and leukotrienes are also released by the NLRP3/Caspase-1 axis^46,47^. NLRP3 inhibitors or genetic deficiency have been shown to decrease the production of various inflammatory cytokines, including TNF-α, IL-6, IL-1β, MIP-2 and lipid mediators, and provide protection against septic lethality^14–16^. Consequently, we measured the production of IL-6, TNF-α and IL-1β in sepsis patients and healthy controls and in PBMCs under in vitro LPS stimulation to investigate the effect of the NLRP3 29940 G>C variation on the production of these related cytokines. It has been reported that the NLRP3 inflammasome is significantly activated due to a gain-of-function alteration in rs35829419 in NLRP3, causing excessive IL-1β, IL-18 and TNF-α production and exaggerated inflammatory responses in a caspase-1-dependent manner^40^. Our results showed a close association between TNF-α production but not IL-1β or IL-6 with the NLRP3 29940 G>C variation in sepsis patients, which might be due to the complex inflammatory system in the host. Furthermore, PBMCs with the sepsis-associated risk allele 29940G in NLRP3 exhibited significantly enhanced expression of NLRP3 and release of IL-1β and TNF-α compared to those with the C allele under LPS stimulation. These results further confirmed that the 29940 G-to-C mutation in the NLRP3 3′-UTR suppressed NLRP3 expression and downstream inflammatory cytokine production by binding with miR-146a in a gain-of-function manner, which ultimately protected patients against susceptibility to sepsis progression and exhibited potentially important diagnostic and therapeutic value (Fig. 7).

Several limitations in the present study should be mentioned. The undiscovered underlying confounding factors, including pre-existing conditions in sepsis patients, could have influenced those observed differences in our results, even though exclusion and inclusion criteria were used to obtain more homogeneity. Furthermore, this study was restricted to the Chinese Han population with small sample size. According to the international HapMap project, the frequency of G allele at rs10754558 site in Han Chinese descent (0.41) is similar to those in several populations, such as Finnish in Finland (0.42) and British in England and Scotland (0.41), while it differed from those in other populations, such as Americans of African Ancestry (0.24) and African Carribbeans in Barbados (0.31). Therefore, one should exercise caution when generalizing the findings to other ethnic groups, and these findings need to be further validated in a larger and multiethnic population of subjects.

## Conclusions

The data in this study indicated that the 29940 G>C polymorphism in the 3′-UTR of NLRP3 conferred susceptibility to the development of sepsis and a poor clinical outcome. This 29940 G-to-C mutation was demonstrated to be a functional polymorphism that cause the suppression of NLRP3 transcription and downstream inflammatory cytokine production by binding with miR-146a-5p in a gain-of-function manner, which ultimately conferred protection against progression from sepsis to septic shock. These results may provide a new understanding of the genetic effect of the NLRP3 inflammasome on sepsis pathophysiology and new approaches for risk assessments and therapeutic strategies for sepsis patients.

## Supplementary Information

Supplementary Information.

## Data Availability

All data generated or analysed during this study are included in this published article.

## Abbreviations

NLRP3: Nucleotide-binding domain and leucine-rich repeat (LRR)-containing family protein 3
SNP: Single-nucleotide polymorphism
qRT-PCR: Quantitative real-time polymerase chain reaction
ELISA: Enzyme-linked immunosorbent assay
TNF-a: Tumor necrosis factor alpha
IL-1β: Interleukin-1 beta
IL-6: Interleukin-6
cDNA: Complementary deoxyribonucleic acid
PBMC: Peripheral blood mononuclear cell
HUVEC: Human umbilical vein endothelial cell
LPS: Lipopolysaccharides
QUANTO: Quantity adjusting option
