# Supplementary material for: A gain-of-function NLRP3 3′-UTR polymorphism causes miR-146a-mediated suppression of NLRP3 expression and confers protection against sepsis progression
Source: Sci Rep. 2021 Jun 25;11:13300. doi: 10.1038/s41598-021-92547-8 (PMC8233413; doi:10.1038/s41598-021-92547-8)
Supplement: Supplementary file 1 — Supplementary Information. [file 41598_2021_92547_MOESM1_ESM.pdf]

A gain-of-function NLRP3 3'-UTR polymorphism causes miR-146a-mediated suppression of NLRP3 expression and confers protection against sepsis progression

Author:

Furong Lu[1#], Hongpeng Chen[4#], Yuan Hong[1#], Yao Lin[2], Lizhen Liu[1,3], Ning Wei[1], Qinyan Wu[1], Shuanglin Liao[1], Shuai Yang[1], Junbing He[2#], Yiming Shao[1,3,5]*

Author details:

[1]The Key Laboratory of Sepsis Translational Medicine, The Intensive Care Unit, The Second Affiliated Hospital of Guangdong Medical University, Zhanjiang, Guangdong, PR China

[2] The Clinical Medicine Research Laboratory, The Intensive Care Unit, Jieyang Affiliated Hospital, SunYat-sen University, Jieyang, Guangdong, PR China

[3]The Intensive Care Unit, Affiliated Hospital of Guangdong Medical University, Zhanjiang, Guangdong, PR China

[4]The Department of Chemotherapy, Jieyang Affiliated Hospital, SunYat-sen University, Jieyang, Guangdong, PR China

[5]Southern Marine Science and Engineering Guangdong Laboratory (Zhanjiang), Zhanjiang, Guangdong, 524023, China.

[#] These authors contributed equally to this work.

*** Correspondence:**

1. Yiming Shao: E-mail: sym@gdmu.edu.cn; Physical address: The Key Laboratory of Sepsis Translational Medicine, The Intensive Care Unit, The second Affiliated Hospital of Guangdong Medical University, Minyou Road 12, Xiashan district, Zhanjiang City 524001, Guangdong Province, PR China; Phone: +86 18826690188

2. Junbing He: E-mail: junbinghe_gmc@163.com; Physical address: The Intensive Care Unit, Jieyang Affiliated Hospital, SunYat-sen University, Tianfu Road 107, Rongcheng district, Jieyang City 522000, Guangdong Province, PR China; Phone: +86 13612424221

Supplementary Table S1. Genotype distributions.

**rs10754558**	**Patients n (%)**	**Controls n (%)**	**Sepsis subtype n (%)**	**Septic shock n (%)**
GG	105 (16.4)	127 (16.5)	49 (13.8)	56 (19.6)
GC	314 (49.1)	398 (51.8)	166 (46.9)	148 (51.7)
CC	221 (34.5)	244 (31.7)	139 (39.3)	82 (28.7)
Hardy-Weinberg equilibrium test *P* value	0.712	0.098	0.960	0.461

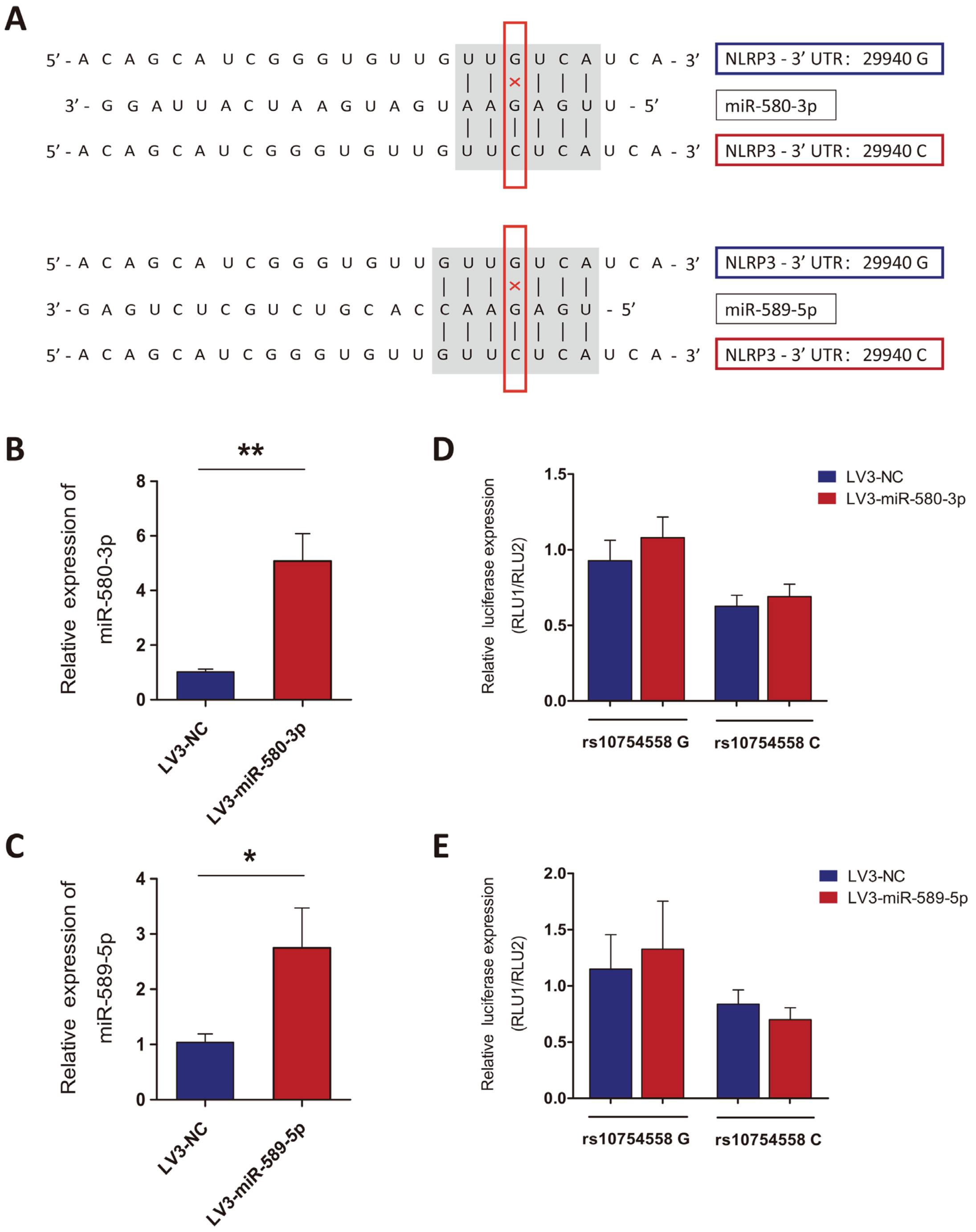

Supplementary Fig. S1. (**A**) Bioinformatics analyses predicted by miRNASNP-v3 and TargetScan revealed a target binding site of miR-580-3p and miR-589-5p enabled by the 29940 G-to-C mutation within the 3'-UTR of the NLRP3 gene; (**B,C**) THP-1 cells were infected by LV3-miR-580-3p (miR-580-3p overexpression), LV3-miR-589-5p (miR-589-5p overexpression) or LV3-NC, and the infection efficiency was determined by qRT-PCR analysis; (**D,E**) The effect of miR-580-3p or miR-589-5p on the transcriptional activities of rs10754558 G>C in THP-1 cells were determined by dual-luciferase report assays. Bar graphs indicate the mean ± SEM for a minimum of three independent experiments. **P*<0.05, ***P*<0.01.

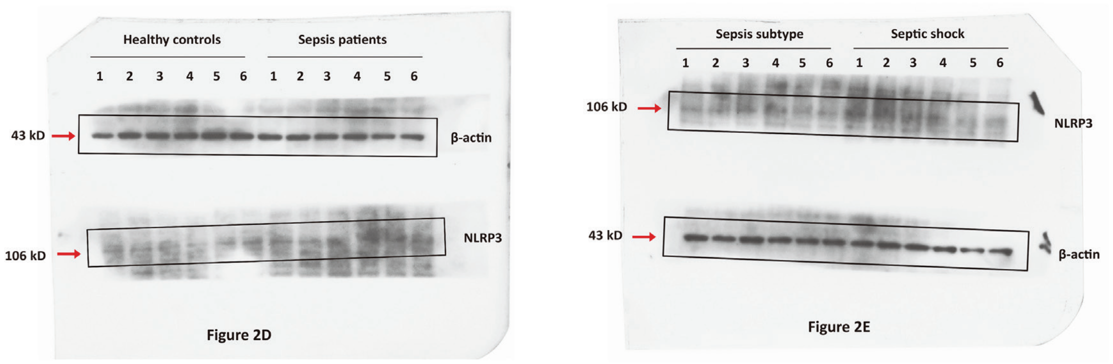

Supplementary Fig. S2. The PBMCs isolated from 18 sepsis patients and 6 healthy controls were used for NLRP3 protein measurement by Western blot analysis. Equal amounts of protein were separated and then transferred onto polyvinylidene difluoride membranes. The membranes were incubated with antibodies against NLRP3 and β-actin at 4°C overnight, followed by HRP-linked secondary antibody. The protein bands were visualized using a BeyoECL Star kit (Beyotime, Shanghai, China). The order of sample loading and antibodies are marked in the up figure, and the blots in the rectangular box were used in the Figure 2D and Figure 2E.
